# Characterization and CRISPR-based genotyping of clinical *trh*-positive *Vibrio parahaemolyticus*

**DOI:** 10.1186/s13099-018-0275-4

**Published:** 2018-11-13

**Authors:** Jetnapang Kongrueng, Kanchana Srinitiwarawong, Mitsuaki Nishibuchi, Pimonsri Mittraparp-arthorn, Varaporn Vuddhakul

**Affiliations:** 1 Department of Microbiology, Faculty of Science, Prince of Songkla University, Hat Yai, Thailand; 2 Center for Southeast Asian Studies, Kyoto University, Kyoto, Japan

**Keywords:** CRISPR, CRISPR-virulence typing, *trh* gene, *Vibrio parahaemolyticus*

## Abstract

**Background:**

*Vibrio parahaemolyticus* is a causative agent of gastroenteritis. Most of the clinical isolates carry either *tdh* and/or *trh* genes which are considered as the major virulence genes of this pathogen. In this study, the clinical isolates of *V. parahaemolyticus* carrying *trh* gene (*n *= 73) obtained from 1886 to 2012 from various countries were investigated for the urease production, haemolytic activity, and biofilm formation. In addition, the potential of clustered regularly interspaced short palindromic repeats (CRISPR)-based genotyping among these isolates was investigated.

**Results:**

In this study, no significant differences were observed in the urease production between *tdh*^+^ *trh*1^+^ and *tdh*^+^ *trh*2^+^ isolates (*p* = 0.063) and between the *tdh*^−^ *trh*1^+^ and *tdh*^−^ *trh*2^+^ isolates (*p* = 0.788). The isolates carrying only the *trh* gene showed variation in their haemolytic activity. The ratio of urease production and haemolytic activity between the *trh*1^+^ and *trh*2^+^ isolates and biofilm formation of *trh*^+^ *V. parahaemolyticus* isolates were not significantly different. Sixteen of thirty-four tested isolates (47.0%) of *trh*^+^ *V. parahaemolyticus* were positive for CRISPR detection. The discriminatory power index (DI) of CRISPR-virulence typing was higher than the DI obtained by CRISPR typing alone.

**Conclusion:**

The *tdh* and *trh* genes were not involved in urease production in the *trh*^+^ *V. parahaemolyticus*, and variation of haemolytic activity detected in *V. parahaemolyticus* carrying only the *trh* gene might be correlated to the sequence variation within *trh*1 and *trh*2 genes. Additionally, biofilm production of *V. parahaemolyticus* was not associated with harboring of virulence genes. For genotyping, CRISPR sequences combined with virulence genes can be used as genetic markers to differentiate *trh*^+^ *V. parahaemolyticus* strains.

**Electronic supplementary material:**

The online version of this article (10.1186/s13099-018-0275-4) contains supplementary material, which is available to authorized users.

## Background

*Vibrio parahaemolyticus* is a halophilic Gram-negative bacterium that occurs naturally in worldwide aquatic environments. The organism causes gastroenteritis in several countries due to consumption of raw or undercooked seafood [1]. The pathogenicity of this bacterium in humans is associated to the production of thermostable direct hemolysin (TDH) encoded by *tdh* gene and/or TDH-related hemolysin (TRH) encoded by *trh* gene, which are considered to be the major virulence factors, however, not many isolates from the environment possess these genes [2]. *V. parahaemolyticus* obtained the *tdh* and *trh* genes from other organisms and transmit them among *V. parahaemolyticus* strains via horizontal gene transfer [3]. TDH and TRH are approximately 67% identity in their amino acid sequences and possess common biological activities including haemolytic activity, enterotoxicity, cytotoxicity, and cardiotoxicity [4]. The *trh* gene possesses a significantly broader nucleotide sequence variation and can be subdivided into two main subtypes (*trh*1 and *trh*2) which share 84% identity in their sequences  [5]. The presence of *trh* gene in *V. parahaemolyticus* isolates is associated with the urease production because of the genetic linkage between *ure* and *trh* genes on the chromosome of *trh*^+^ *V. parahaemolyticus* [6]. Urease is an enzyme that catalyzes the hydrolysis of urea to ammonia, raising the pH of environment inside the host. It is possible that urease contributes to the pathogenicity of this bacterium by increasing its chance of survival after entering the human body, as has been previously demonstrated for *Yersinia enterocolitica* infections [7]. Clustered regularly interspaced short palindromic repeats (CRISPR) and repetitive sequences such as tandem repeats are commonly detected in the genomes of bacteria. Their lengths and numbers are highly variable among bacterial strains and are useful for bacterial genotyping [8]. CRISPR is a segment of prokaryotic DNA containing direct repeats (DRs), typically 24–47 bp nucleotides in length, and spacer (non-repetitive sequences obtained from foreign genetic elements). The polymorphism detected in CRISPR loci has been applied as a genetic marker for typing many bacteria, such as *Campylobacter fetus* and *S. Typhimurium* [9, 10]. Association of CRISPR and virulence factors of bacteria has been demonstrated in *Pseudomonas aeruginosa*, *Myxococcus xanthus*, *Francisella novicida* and *Listeria monocytogenes* [11]. In *V. parahaemolyticus*, correlation between CRISPR and virulence genes was determined in 208 isolates from clinical and food samples [12]. CRISPR was detected in 149 of 153 (97.4%) of *tdh*^+^ isolates. In contrast, among the 6 *trh*^+^ *V. parahaemolyticus* tested isolates, only 2 were positive for CRSIPR locus [12]. Therefore, association of CRISPR and the *trh*^+^ *V. parahaemolyticus* is not completely concluded.

In this work, *trh*^+^ *V. parahaemolyticus* isolates from clinical samples were investigated for urease production, haemolytic activity and biofilm formation. In addition, molecular typing based on CRISPR was analyzed.

## Methods

### Bacterial strains

A total of 73 clinical *trh*^+^ *V. parahaemolyticus* isolates were obtained from various countries between 1886 and 2012 (Table 1). They belonged to 28 different serotypes consisting of 10 O serogroups and 19 K antigens. All isolates were confirmed as *V. parahaemolyticus* using PCR targeted to the *toxR* gene [13].

**Table 1:** A total of 73 isolates of *trh*-positives *V. parahaemolyticus* isolated from clinical samples

Country	Year	Presence of gene *tdh trh*1 *trh*2			No. of isolates	O:K serotype (no. of isolates)
Thailand	1991–2012	+	+	–	14	O1:KUT (3), O3:K6 (5), O3:K72 (2), O4:K62 (2), O4:K63 (1), O12:KUT (1)
	1987–2006	–	+	–	10	O1:K48 (1), O1:K56 (1), O1:K69 (1), O3:K6 (3), O3:KUT (2), O4:K53 (1), O5:KUT (1)
	1886–2012	+	–	+	9	O1:K1 (1), O1:KUT (4), O1:K69 (1), O3:K72 (1), O3:KUT (1), O8:K56 (1)
	1999–2012	–	–	+	6	O1:K25 (1), O1:K41 (2), O1:K69 (1), O1:KUT (2)
USA	1990–1996	+	+	–	5	O1:K56 (1), O4:K12 (2), O4:K63 (1), O1:KUT (1)
	1990	–	+	–	1	O4:K12 (1)
	1991–1996	–	–	+	2	O3:K59 (1), O11:K15 (1)
Bangladesh	1994	+	+	–	1	O4:K11 (1)
	1981	–	+	–	1	O4:K11 (1)
	1977–1986	–	–	+	11	O1:K25 (1), O1:K56 (2), O1:KUT (3), O3:K7 (1), O3:KUT (2), O13:KUT (1), O5:KUT (1)
Maldives	1985	–	+	–	1	O3:K6 (1)
Singapore	1985–1992	+	–	+	2	O1:K69 (1), O1:KUT (1)
	1985	–	+	–	1	O4:K11 (1)
Vietnam	2010	–	+	–	1	O1:K1 (1)
India	1994	+	–	+	1	O1:KUT (1)
	1994	–	–	+	1	O1:KUT (1)
Philippines	1983–1987	+	–	+	3	O3:KUT (1), O10:K71 (1), O6:K46 (1)
Hong Kong	1983–1993	+	–	+	2	O1:K1 (1), O1:K69 (1)
Malaysia	1995	+	–	+	1	O4:K12 (1)

### Detection of virulence genes

Genomic DNA of all *V. parahaemolyticus* isolates was extracted using boiling method [14] and was used as templates to detect the virulence genes, *tdh*, *trh*1 and *trh*2. A 251-bp sequence of *tdh* gene was amplified by PCR [15]. For detection of *trh* genes, PCR was performed using two primer sets based on *trh*1 and *trh*2 sequences available in the NCBI GenBank database. The *trh*1 primers: Trh1-F1 (5ʹ-CTGAATCACCAGTTAACGC-3ʹ) and Trh1-R1: (5ʹ-GGCGTTTRATCCAAATAC-3ʹ) generated a PCR product of 313-bp and *trh*2 primers: Trh2-F2 (5ʹ-CAATCAAAACTGAATCCCC-3ʹ) and Trh2-R3 (5ʹ-CATCAACAAAAMATTTTACCGA-3ʹ) provided an amplicon of 276-bp. The PCR reaction was carried out with a reaction mixture consisting of 1.5 mM MgCl_2_, 0.2 mM dNTPs, 0.2 μM of each primer, 0.025 U of *GoTaq* DNA polymerase and 2.0 μl DNA templates in a 20 μl volume. The reactions were performed with a Thermal Cycler Gene Atlas (Astec, Fukuoka, Japan) as follows: 5 min for a hot start at 96 °C, followed by 35 cycles of amplification consisting of denaturation at 94 °C for 1 min, annealing at 53 °C for 1 min and extension at 72 °C for 1 min and final extension at 72 °C for 7 min. Electrophoresis was performed on a 1.5% agarose gel and the amplicons were detected using a UV transilluminator. In addition, the specificity of the *trh*1 and *trh*2 primers was also determined by purification of PCR products using ethanol/sodium acetate precipitation, and sequencing.

### Quantitative urease assay

Urease was quantified using a colorimetric assay based on the reaction of ammonia (NH_3_) and phenol in the presence of hypochlorite which yields a blue product of indophenol [16]. Briefly, bacteria were inoculated in LB broth supplemented with 3% sodium chloride (NaCl) and 0.1% urea and incubated at 37 °C. Overnight cultures were concentrated by centrifugation, washed twice with 50 mM HEPES buffer (pH 7.5) and resuspended in the same buffer. The cells were lysed by sonication, then, 50 μl of supernatant obtained after centrifugation was mixed with 25 mM of urea in HEPES buffer (pH 7.5). After incubation at 37 °C for 30 min, ammonia released from the lysate was determined by adding solution of 1% (w/v) phenol and 170 μM sodium nitroprusside followed by solution of 125 mM sodium hydroxide (NaOH) and 0.05% (w/v) sodium hypochlorite (NaOCl). Subsequently, the tubes were incubated at 37 °C for 30 min and the absorbance was determined on a Hitachi U 2000 Double-Beam UV/VIS spectrophotometer (Hitachi Instruments Inc., Danbury, CT) at a wavelength of 625 nm. Ammonium chloride (NH_4_Cl) was used to perform standard curve. The total protein concentration from the same lysate was determined by Lowry’s method with Folin-Ciocalteu’s reagent solution (Nacalai Tesque Inc., Kyoto, Japan) and bovine serum albumin (BSA) was used to set up standard curve [17]. Urease activity was calculated as micromoles of NH_3_ per minute per milligram of protein.

### Determination of haemolytic activity

*V. parahaemolyticus* carrying either the *trh*1 or *trh*2 gene could lyse human erythrocyte [5, 18]. In this study, haemolytic activity of 15 *tdh*^**−**^ *trh*1^+^ and 20 *tdh*^**−**^ *trh*2^+^ isolates of *V. parahaemolyticus* was evaluated using the blood agarose assay [19] with some modification. Briefly, *V. parahaemolyticus* was grown in LB broth supplemented with 2% NaCl at 37 °C for 18 h, the pellet was harvested and resuspended in PBS (pH 7.0). Then, the cells were sonicated and the supernatant obtained by centrifugation was determined. The 50 μl of the supernatant was added into the agarose wells containing various concentration of human blood (1%, 0.5% and 0.25%). After incubation at 37 °C for 24 h, a clear zone around the well was indicated as haemolytic activity. High haemolytic activity (+3) was defined as the isolates can lyse all three concentration of erythrocytes whereas medium (+2) and low (+1) haemolytic activities were defined as the isolates can haemolyse two (0.5% and 0.25%) and one (0.25%) blood concentrations, respectively.

### Quantitative biofilm assays

Each 5 isolates of *V. parahaemolyticus* belonging to the *tdh*^**−**^ *trh*1^+^, *tdh*^**−**^ *trh*2^+^, *tdh*^**+**^ *trh*1^+^ and *tdh*^**+**^ *trh*2^+^ isolates was investigated for biofilm formation as previously described [20]. Biofilm formation was quantified by measuring the optical density (OD) at 570 nm using a LUMIstar Omega spectrophotometer (BMG LABTECH, Germany). LB broth supplemented with 2% NaCl was used as control. The experiment was performed in triplicate.

### Determination of CRISPR using PCR technique

The primers for detection of CRISPR of *V. parahaemolyticus* were designed from the CRISPR sequences of *V. parahaemolyticus* serotype O3:K6 (strain RIMD2210633) obtained from the CRISPR database website (http://crispr.u-psud.fr/) [21]. The forward primer: VpCRISPR_3-F (5ʹ-ATGCATTCCAAAGCTACCACTC-3ʹ) and the reverse primer: VpCRISPR_705-R (5ʹ-GCCTACCAGATAGCAAGTGTCC-3ʹ) generated a 592-bp product. The PCR reaction mixture consisted of 1 × *Ex Taq* Buffer, 2 mM MgCl_2_, 1.25 U Takara *Ex Taq* DNA polymerase (Takara Biochemicals, Tokyo, Japan), 200 μM dNTPs, 0.2 μM of each primer, and 5 μl of DNA template in a total volume of 50 μl. The PCR reaction was performed using the following conditions: initial denaturation cycle for 1 min at 94 °C, followed by 30 cycles of amplification consisting of denaturation at 94 °C for 1 min, annealing at 50 °C for 1 min and extension at 72 °C for 1 min and final extension at 72 °C for 5 min. The PCR product was confirmed by sequencing using the forward primer: VpCRISPR_3-F.

CRISPR pattern including the DRs and spacers was investigated using the CRISPRfinder tool (http://crispr.i2bc.paris-saclay.fr/Server/). The DR sequences in each isolate were analyzed based on the similarity of consensus direct repeat sequences (CDRs). In addition, all spacer sequences were investigated using the CRISPRTarget tool (http://bioanalysis.otago.ac.nz/CRISPRTarget/) and were used for phylogenetic tree construction.

In this work, CRISPR-virulence typing was constructed and compared to profiles obtained by CRISPR typing alone. CRISPR-virulence typing was constructed based on the CRISPR spacer sequences and the presence of virulence genes that included the *tdh*, *trh*1 and *trh*2 genes. A profile of each isolate was created using a binary matrix of presence or absence of spacer sequences and virulence genes. The dendrogram was constructed using BioNumerics 7.0 software (Applied Maths, Saint-Martens-Latem, Belgium) with the UPGMA algorithm using the Dice similarity coefficient. The discriminatory power index (DI) of CRISPR typing alone and CRISPR-virulence typing were assessed by Simpson’s diversity index [22].

### Statistical analysis

Urease activity and correlation between urease and haemolytic activities of *trh*^+^ *V. parahaemolyticus* isolates were determined by the independent samples *t* test analysis using SPSS 11.5 software. The differences observed were considered statistically significant at *p* < 0.05.

## Results and discussion

### Urease production in *trh*^+^*V. parahaemolyticus*

**Fig. 1:**
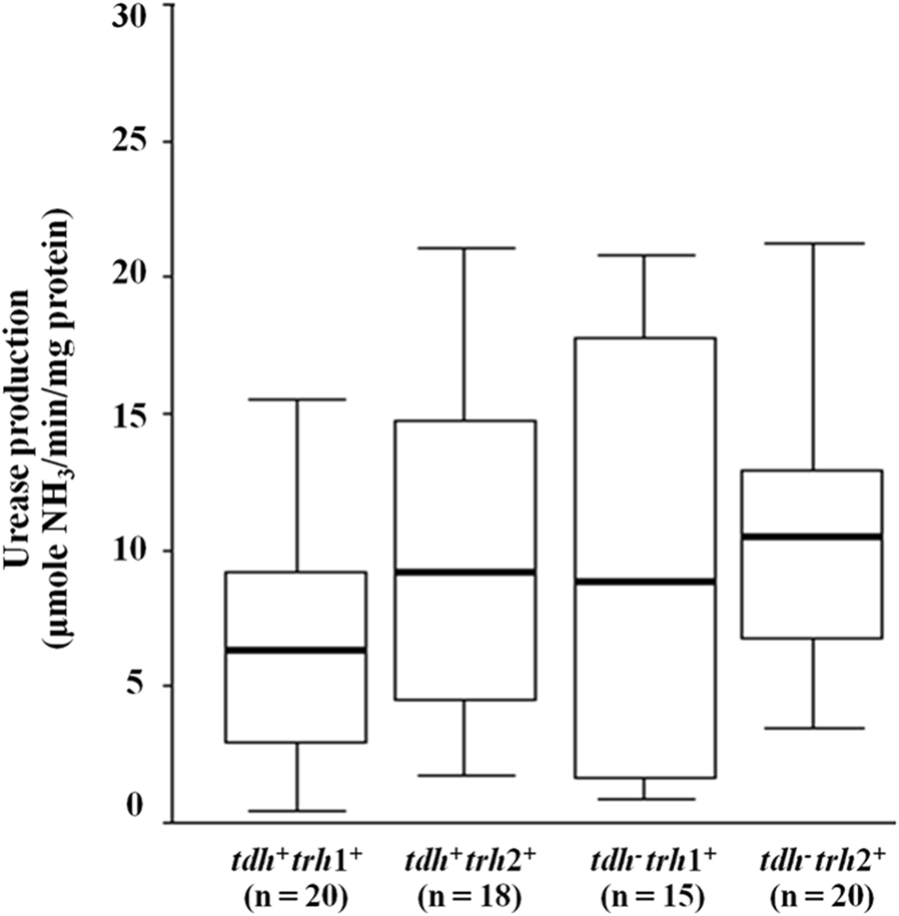
Comparison of urease production between *trh*^+^ *V. parahaemolyticus* isolates according to the presence or absence of *tdh*. Horizontal bar within box represents median values and vertical line out of the box indicates minimum and maximum. The difference in urease production in each group was compared using the independent samples *t*-test analysis

Urease is detected in many pathogenic bacteria such as *Proteus mirabilis*, *Helicobacter pylori* and *Yersinia enterocolitica* [23–25]. In *V. parahaemolyticus*, urease-positive phenotype strongly correlates with the possession of the *trh* gene making it as a marker of *trh*^+^ strains [26]. In this work, urease production in *trh*^+^ *V. parahaemolyticus* isolates was evaluated. Previous results have suggested that the presence of urea is involved in the urease induction of *trh*^+^ *V. parahaemolyticus* TH3996 strain [27]. Therefore, in this work, all tested isolates were grown in the liquid medium supplemented with 0.1% urea before testing. Urease production of the *tdh*^+^ *trh*1^+^*, tdh*^+^ *trh*2^+^*, tdh*^−^ *trh*1^+^and *tdh*^−^ *trh*2^+^ *V. parahaemolyticus* isolates varied from 0.46 to 15.54, 1.75 to 21.05, 0.89 to 20.79 and 3.46 to 21.20 μmol NH_3_/min/mg protein, respectively. No significant differences were observed in the urease production between the *tdh*^+^ *trh*1^+^ and *tdh*^+^ *trh*2^+^ (*p* = 0.063) as well as in the *tdh*^−^ *trh*1^+^ and *tdh*^−^ *trh*2^+^ isolates (*p* = 0.788) (Fig. 1). These indicated that urease production among clinical *trh*^+^ *V. parahaemolyticus* isolates varied distinctively but it was not associated with either the *trh* or *tdh* genes. In addition, no correlation between urease production and serotypes of *V. parahaemolyticus* was observed in this study (Additional file 1: Table S1).

### Haemolytic activity of *trh*^+^*V. parahaemolyticus*

**Fig. 2:**
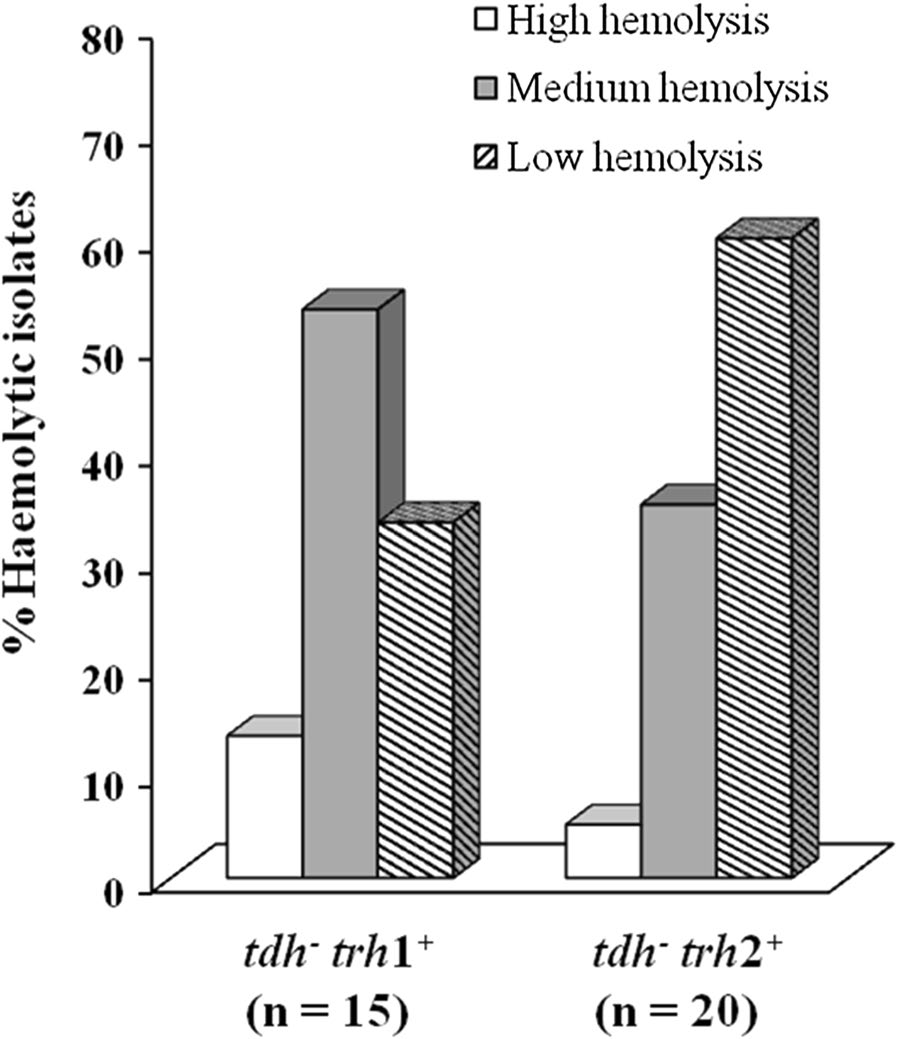
Haemolytic activity of *V. parahaemolyticus* carrying only the *trh* gene isolated from clinical samples using blood agarose assay

A total of 15 *trh*1^+^ isolates was determined, 13.3%, 53.3% and 33.3% of the total isolates exhibited high, medium and low haemolytic activity, respectively (Fig. 2). For 20 isolates of *V. parahaemolyticus* carrying the *trh*2 gene, 5%, 35% and 60% of the total isolates displayed high, medium and low haemolytic activity. It has been demonstrated that the expression of *trh*2 was lower than the *trh*1 [5, 28]. Therefore, the results obtained in this study might correlate to the expression of the genes and the sequences variation within the *trh*1 and *trh*2 genes [5].

**Fig. 3:**
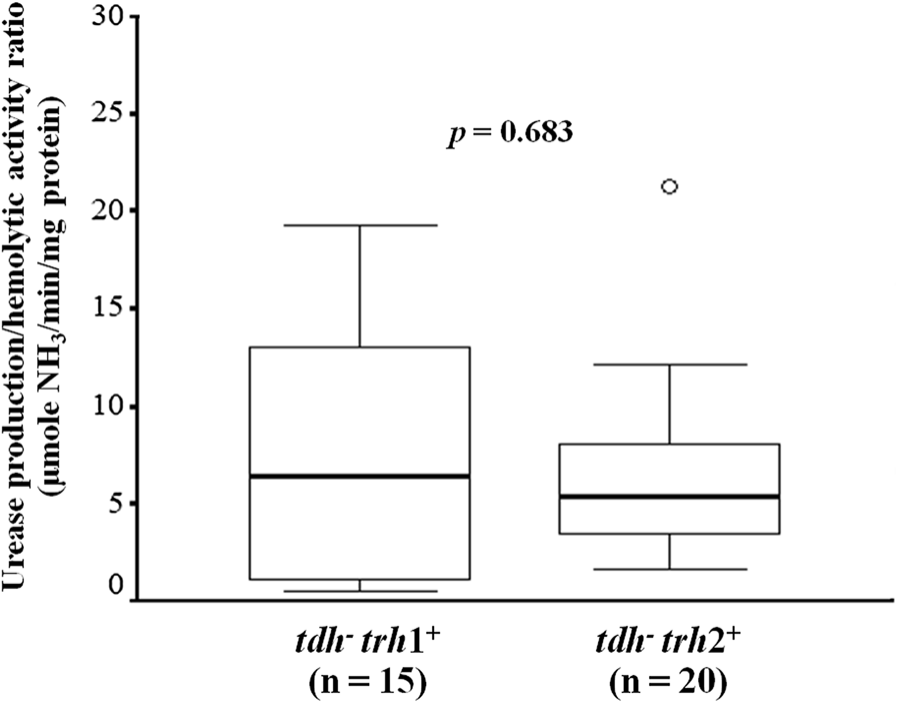
Correlation between urease production and haemolytic activity of *V. parahaemolyticus* isolates carrying only the *trh* gene. Horizontal bar within box represents median values and vertical lines out of the box indicate minimum and maximum. ◯ indicates the outlier. No significant differences were observed in the urease production/haemolytic activity between the isolates carrying the *trh*1 and *trh*2 genes (*p* = 0.683) using the independent samples *t*-test analysis

In this work, correlation between urease production and haemolytic activity of *trh*^+^ isolates was evaluated. Although the urease production and haemolytic activity ratio of *trh*2^+^ isolates was higher (1.65 to 21.20 μmol NH_3_/min/mg protein) than that of the *trh*1^+^ isolates (0.56–19.21 μmol NH_3_/min/mg protein), no significant difference was observed (*p* = 0.683) (Fig. 3). This indicated the urease was not involved in the human erythrocyte lysis by TRH hemolysin of *trh*^+^ *V. parahaemolyticus* strains.

### Biofilm formation

**Fig. 4:**
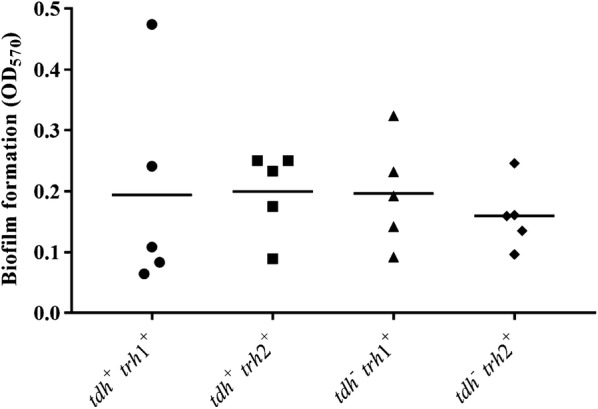
Biofilm formation of *trh*^+^ *V. parahaemolyticus* isolated from clinical samples. Symbols represent mean of biofilm formation of each isolates and horizontal bar indicate mean of biofilm formation of each category

Most of human bacterial infections are associated with biofilms that contributed to their resistance and persistence in the host [29]. Previous study demonstrated that biofilm formation of vibrios was a survival mechanism associated with their pathogenesis and stress tolerance [30]. In order to investigate the involvement of the virulence genes and biofilm production, 4 categories of *V. parahaemolyticus* (*tdh*^**+**^ *trh*1^+^, *tdh*^**+**^ *trh*2^+^*, tdh*^**−**^ *trh*1^+^, and *tdh*^**−**^ *trh*2^+^) were determined. There was no any difference in biofilm formation among 4 categories of *V. parahaemolyticus* because high variation of biofilm formation within the isolates in each category was observed (Fig. 4). Variation in biofilm formation among the bacterial isolates has been reported. High variability in biofilm formation of 34 strains of *Acinetobacter baumannii* isolated from hospitalized patients was observed without correlation to molecular types and antimicrobial resistance [31]. Ninety-eight strains with the same serotype of *Listeria monocytogenes* displayed different biofilm formation [32]. Additionally, quantitative biofilm assay of *Vibrio cholerae* isolates in Thailand demonstrated that those isolates possessed a wide range of biofilm production [33].

### CRISPR sequences in *trh*^+^*V. parahaemolyticus*

**Fig. 5:**
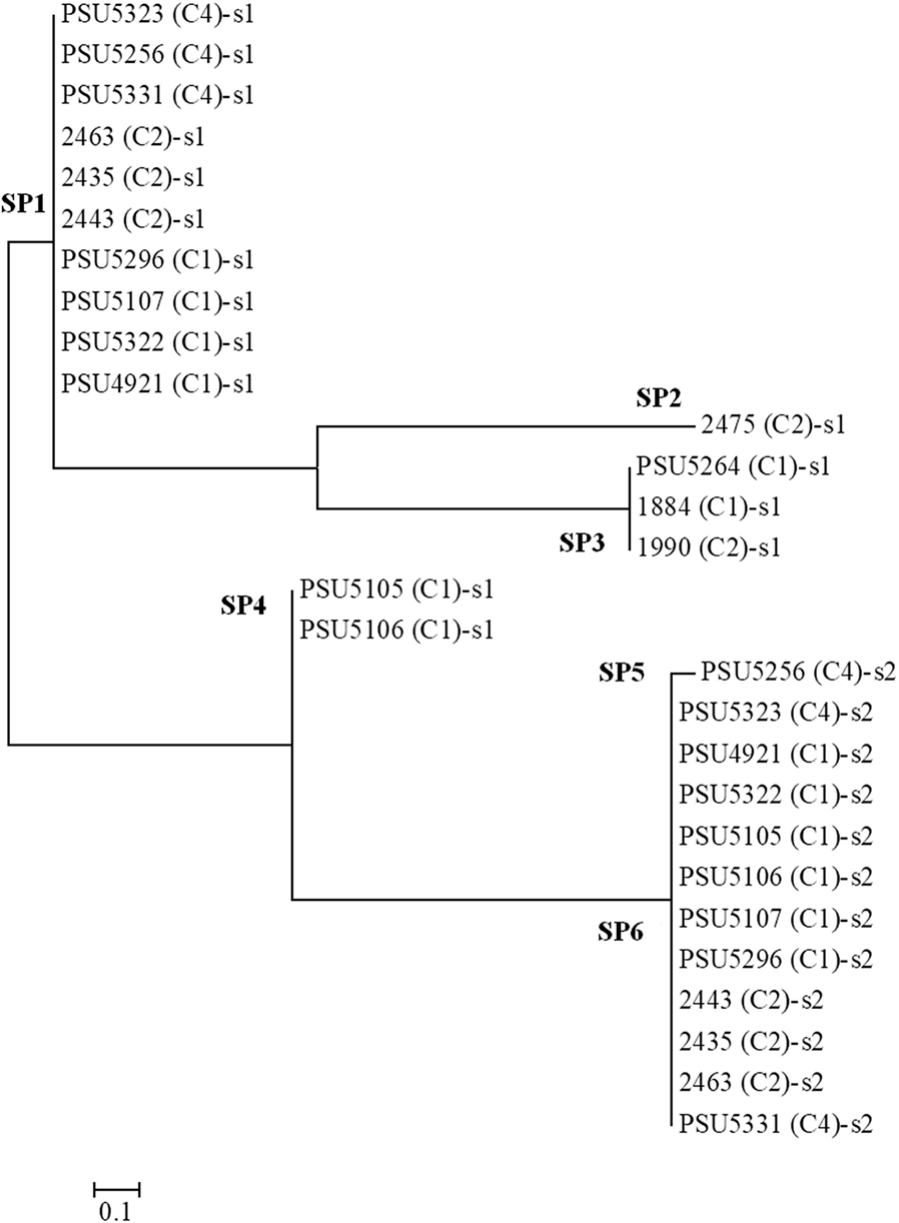
Phylogenetic relationship of all 28 spacer sequences detected in 16 CRISPR-positive *V. parahaemolyticus* isolates. The tree was constructed using maximum likelihood method. Numbers at branch-points represent confidence values obtained after bootstrap analysis of the maximum likelihood tree using 1000 replicates. The scale bar represents 0.1 substitutions per nucleotide position. PSU5256-s2, spacer no. 2 of *V. parahaemolyticus* PSU5256; C1, *tdh*^+^ *trh*1^+^; C2*, tdh*^+^ *trh*2^+^; C3, *tdh*^−^ *trh*1^+^; C4, *tdh*^−^ *trh*2^+^

In the present study, specific primer pairs for detecting CRISPR region were designed and compared to the previously reported by Sun and co-workers [12]. No difference in the specificity detected in the 5 *V. parahaemolyticus* tested isolates after confirmation by sequencing (data not shown). Thirty-four isolates of *trh*^+^ *V. parahaemolyticus* including 10 isolates of *tdh*^+^ *trh*1^+^ and each 8 isolates of *tdh*^+^ *trh*2^+^*, tdh*^−^ *trh*1^+^*, tdh*^−^ *trh*2^+^ were selected for CRISPR sequences analysis. Sixteen isolates (47.0%) were positive for CRISPR, they were 8, 5 and 3 from *tdh*^+^ *trh*1^+^, *tdh*^+^ *trh*2^+^, and *tdh*^−^ *trh*2^+^ isolates, respectively (Table 2). None of the isolate carrying only the *trh*1 gene was positive for CRISPR. The result indicated that the number of CRISPR-positive isolates of *trh*^+^ *V. parahaemolyticus* obtained in this study was higher than the 2 out of 6 (33.3%) isolates reported in a previous study [12]. It is of interest that CRISPR was mostly detected in the isolates carrying the *trh* gene together with the *tdh* gene. Association of *tdh* and the presence of CRISPR was observed in 97.4% of *V. parahaemolyticus* isolates [12]. Thus, horizontal gene transfer of virulence genes might have occurred among *V. parahaemolyticus* and close related species. The numbers of DRs were between 25 and 28 bp nucleotides in lengths and the DR unique sequences of all CRISPR-positive isolates were GTGAACTGCCGAATAGGTAGCTGAT (Table 2). A total of 28 spacers were obtained and the number of spacer detected in each isolate was between 1 or 2 with 30 to 32 bp nucleotides in lengths (Additional file 1: Table S1). Spacer analysis using the CRISPRTarget and the BLAST databases from NCBI revealed that most of them showed 87–100% similarity to *Vibrio alginolyticus* plasmids except one spacer of PSU5256 suggesting the possibility of genetic transfer between *V. alginolyticus* and *V. parahaemolyticus* (Additional file 2: Table S2). Phylogenetic tree of all 28 spacers were generated and 6 different spacer patterns designated as SP1 to SP6 were classified using maximum likelihood method (Fig. 5).

**Table 2:** Characteristics of CRISPR loci in all 16 CRISPR-positive *V. parahaemolyticus* isolates

Isolate assigned	Gene harboring	Consensus direct repeats (CDRs) sequences^a^	No. of DRs	No. of spacers	CRISPR locus pattern (bp)^b^
PSU4921	*tdh*^+^ *trh*1^+^	GTGAACTGCCGAATAGGTAGCTGATAAT	3	2	28-**31**-28-**31**-28
PSU5105	*tdh*^+^ *trh*1^+^	GTGAACTGCCGAATAGGTAGCTGATAAT	3	2	28-**31**-28-**31**-28
PSU5106	*tdh*^+^ *trh*1^+^	GTGAACTGCCGAATAGGTAGCTGATAAT	3	2	28-**31**-28-**31**-28
PSU5107	*tdh*^+^ *trh*1^+^	GTGAACTGCCGAATAGGTAGCTGATAAT	3	2	28-**31**-28-**31**-28
PSU5264	*tdh*^+^ *trh*1^+^	GTGAACTGCCGAATAGGTAGCTGATAAT	2	1	28-**33**-28
PSU5296	*tdh*^+^ *trh*1^+^	GTGAACTGCCGAATAGGTAGCTGATAAT	3	2	28-**31**-28-**31**-28
PSU5322	*tdh*^+^ *trh*1^+^	GTGAACTGCCGAATAGGTAGCTGATAAT	3	2	28-**30**-28-**31**-28
1884	*tdh*^+^ *trh*1^+^	GTGAACTGCCGAATAGGTAGCTGATA	2	1	26-**31**-26
1990	*tdh*^+^ *trh*2^+^	GTGAACTGCCGAATAGGTAGCTGATAAT	2	1	28-**31**-28
2475	*tdh*^+^ *trh*2^+^	GTGAACTGCCGAATAGGTAGCTGATAAT	2	1	28-**32**-28
2435	*tdh*^+^ *trh*2^+^	GTGAACTGCCGAATAGGTAGCTGATAAT	3	2	28-**32**-28-**31**-28
2443	*tdh*^+^ *trh*2^+^	GTGAACTGCCGAATAGGTAGCTGATAAT	3	2	28-**31**-28-**31**-28
2463	*tdh*^+^ *trh*2^+^	GTGAACTGCCGAATAGGTAGCTGAT	3	2	25-**31**-25-**31**-25
PSU5256	*tdh*^−^ *trh*2^+^	GTGAACTGCCGAATAGGTAGCTGATAAT	3	2	28-**32**-28-**31**-28
PSU5323	*tdh*^−^ *trh*2^+^	GTGAACTGCCGAATAGGTAGCTGATAAT	3	2	28-**30**-28-**31**-28
PSU5331	*tdh*^−^ *trh*2^+^	GTGAACTGCCGAATAGGTAGCTGATAAT	3	2	28-**30**-28-**31**-28

^a^Underline indicates the consensus sequence

^b^Underline indicates the direct repeat length and bold indicates the spacer length

**Fig. 6:**
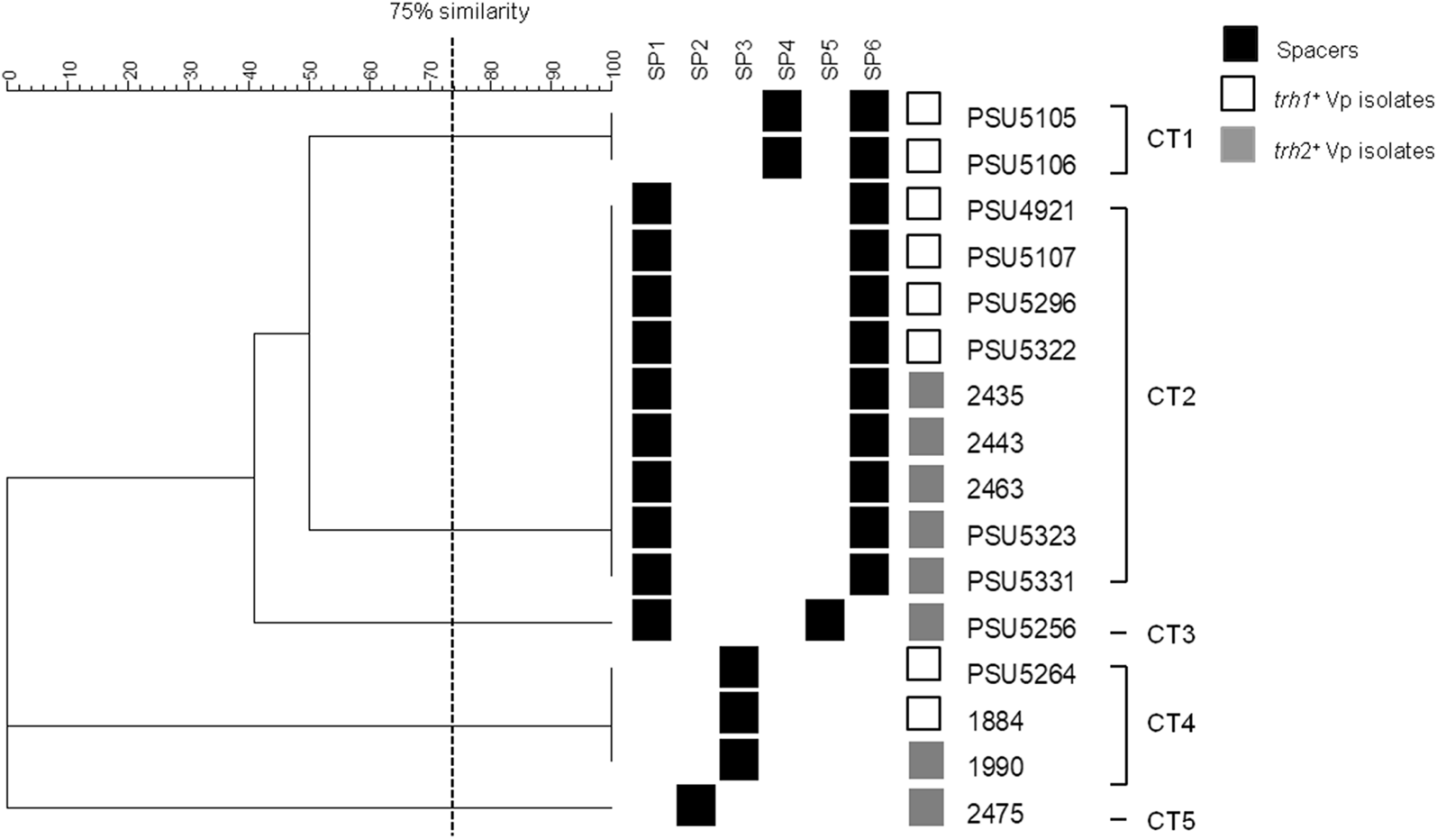
CRISPR–based typing of 16 CRISPR-positive *V. parahaemolyticus* isolates. Dendrogram was constructed based on binary matrix using BioNumerrics 7.0. Similarity (%) between patterns was calculated using the Dice index. The data were sorted using the UPGMA method

CRISPR analysis based on the CRISPR spacer sequences has been applied for bacterial subtyping such as *Campylobacter jejuni*, *Mycobacterium tuberculosis*, *Salmonella enterica* and *Yersinia pestis* [9, 34–36]. In this work, a total of 16 of 34 *trh*^+^ *V. parahaemolyticus* isolates were positive for CRISPR detection, and they were classified into 5 CRISPR types (CTs) based on CRISPR spacer patterns at 75% similarity level (Fig. 6). All isolates in CT1 type were *trh*1^+^ that contained spacers SP4 and SP6. CT2 of both the *trh*1^+^ and *trh*2^+^ isolates harbored spacers SP1 and SP6. One isolate of *trh*2^+^ *V. parahaemolyticus* containing spacers SP1 and SP5 was classified into CT3 type. However, CT4 and CT5 contained only one spacer (SP3 in CT4; SP2 in CT5) (Fig. 6). Although the tested *V. parahaemolyticus* isolates were classified using CPISPR-based typing, they could not be grouped according to the *trh* gene harboring (*trh*1 or *trh*2) (Fig. 6).

**Fig. 7:**
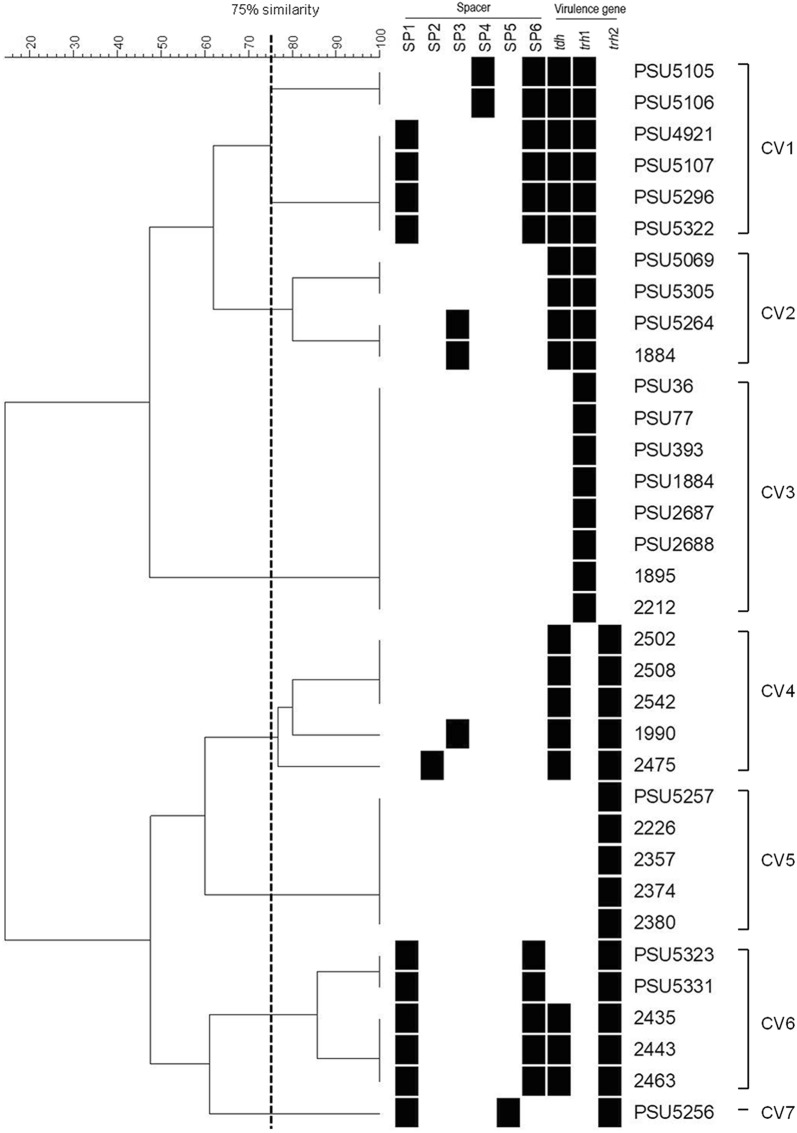
CRISPR-virulence analysis of 34 *trh*^+^ *V. parahaemolyticus*. Dendrogram was constructed based on binary matrix using BioNumerrics 7.0. Similarity (%) between patterns was calculated using the Dice index. The data were sorted using the UPGMA method

Previous study demonstrated that a combination of CRISPR and virulence genes significantly increased the discriminatory power and could be a useful subtyping method for investigation of *Salmonella* outbreaks [37]. In this work, a combination of CRISPR spacer sequences with virulence genes (*tdh*, *trh*1 and *trh*2 genes) of *V. parahaemolyticus* isolates was investigated. CRISPR-virulence typing profiles obtained from 34 isolates of *trh*^+^ *V. parahaemolyticus* were organized into 7 clusters with 12 different profiles at 75% similarity level (Fig. 7). The isolates within the same cluster possessed the identical subtype of the *trh* gene (either *trh*1 or *trh*2). All *trh*1^+^ isolates were classified in the CV1, CV2 and CV3 clusters of CRISPR-virulence typing, while the remaining four clusters (CV4 to CV7) were *trh*2^+^ isolates. The isolates in clusters CV1, CV2, CV4 and CV6 possessed the *tdh* gene, but not all of them gave positive results for CRISPR detection. The CV3 and CV5 clusters were negative for CRISPR (Fig. 7). Identical CRISPR-virulence typing profiles were detected in clusters CV1 (PSU5105 and PSU5106; PSU4921, PSU5107, PSU5296 and PSU5322), CV2 (PSU5069 and PSU5305; PSU5264 and 1884) and CV6 (PSU5323 and PSU5331; 2435, 2443 and 2463) (Fig. 7). It is postulated that these bacteria might obtain the plasmids or exogenous genetic elements derived from the same origin. It was of interest that the spacers SP2 and SP5 were not detected in *trh*1^+^ isolates, whereas *trh*2^+^ isolates lacked spacer SP4 (Fig. 7). The spacer SP2 and SP4 were homologous to *V. alginolyticus* plasmids, whereas spacer SP5 showed no sequence homology with any bacteriophages or plasmids (Additional file 2: Table S2). Self-derived spacers have been detected in some microbial genomes [38–40]. It has been postulated that some bacterial spacers may be self-targeting spacers that are a form of autoimmunity [40, 41].

The discriminatory power index (DI) of both CRISPR analysis and CRISPR-virulence typing were evaluated. DI of CRISPR-virulence typing (DI = 0.90) was higher than that of CRISPR typing (DI = 0.67). This was in agreement with a recent study that DI of CRISPR-virulence typing of *Helicobacter pylori* was higher than CRISPR typing alone [42]. Although, pulse field gel electrophoresis (PFGE) is a good method for discrimination of *V. parahaemolyticus*, it is laborious, time consuming [43, 44]. In this study, Analysis of CRISPR spacers and virulence genes were evaluated for typing *trh*^+^ *V. parahaemolyticus*. CRISPR-virulence gene typing is a method based on PCR detection that provided high DI (0.90). It is easy to perform which required only PCR machine, therefore, CRISPR-virulence gene typing could be a useful method for typing *trh*^+^ *V. parahaemolyticus*.

## Conclusions

A total of 73 isolates of *trh*^+^ *V. parahaemolyticus* obtained from clinical samples were characterized. There was no significant difference in the urease production between the *tdh*^+^ *trh*1^+^ and *tdh*^+^ *trh*2^+^ and between the *tdh*^−^ *trh*1^+^ and *tdh*^−^ *trh*2^+^ isolates indicating that the *tdh* and *trh* genes were not involved in urease production in the *trh*^+^ *V. parahaemolyticus* isolates. The haemolytic activity of *trh*1^+^ isolates was higher than the *trh*2^+^ isolates. Variation in biofilm production was detected in the isolates belonging to the *tdh*^**+**^ *trh*1^+^, *tdh*^**+**^ *trh*2^+^*, tdh*^**−**^ *trh*1^+^, and *tdh*^**−**^ *trh*2^+^ groups. For genotyping, combination of CRISPR spacers and virulence genes provide high discriminatory power than that of CRISPR typing alone and it was able to distinguish between *trh*1^+^ and *trh*2^+^ *V. parahaemolyticus* isolates. Thus, CRISPR-virulence gene typing can be a useful method for typing *trh*^+^ *V. parahaemolyticus* strains.

## Additional files

**Additional file 1: Table S1.** Urease production, haemolytic activity and the ratio of urease production to haemolytic activity of all 73 *trh*^*+*^ *V. parahaemolyticus* isolates.

**Additional file 2: Table S2.** Foreign genetic element similar to spacers using CRISPR targets analysis.
