# Supplementary material for: Characterization and CRISPR-based genotyping of clinical trh-positive Vibrio parahaemolyticus
Source: Gut Pathog. 2018 Nov 13;10:48. doi: 10.1186/s13099-018-0275-4 (PMC6233571; doi:10.1186/s13099-018-0275-4)
Supplement: Supplementary file 1 — Additional file 1: Table S1. Urease production, haemolytic activity and the ratio of urease production to haemolytic activity of all 73 trh+ V. parahaemolyticus isolates. [file 13099_2018_275_MOESM1_ESM.docx]

Additional file 1

**Table S1 Urease production, haemolytic activity and the ratio of urease production to haemolytic activity of all 73 *trh^+^* *V. parahaemolyticus* isolates**

| Isolates | No. of isolates | Isolate assigned | Serotype | Year | Country | UP^a^ | HA^b^ | UP/HA^c^ |
| --- | --- | --- | --- | --- | --- | --- | --- | --- |
| *tdh^+^ trh*1*^+^* | 1 | PSU4921 | O3:K6 | 2010 | Thailand | 13.82 | ND^d^ | ND |
| (n=20) | 2 | PSU5069 | O3:K72 | 2011 | Thailand | 6.88 | ND | ND |
|  | 3 | PSU5090 | O3:K72 | 2011 | Thailand | 6.22 | ND | ND |
|  | 4 | PSU5105 | O1:KUT | 2011 | Thailand | 9.34 | ND | ND |
|  | 5 | PSU5106 | O1:KUT | 2011 | Thailand | 8.96 | ND | ND |
|  | 6 | PSU5107 | O1:KUT | 2011 | Thailand | 6.47 | ND | ND |
|  | 7 | PSU5194 | O3:K6 | 2012 | Thailand | 3.85 | ND | ND |
|  | 8 | PSU5264 | O4:K62 | 2012 | Thailand | 15.54 | ND | ND |
|  | 9 | PSU5268 | O4:K62 | 2012 | Thailand | 2.21 | ND | ND |
|  | 10 | PSU5296 | O4:K63 | 2012 | Thailand | 11.45 | ND | ND |
|  | 11 | PSU5305 | O3:K6 | 2012 | Thailand | 7.43 | ND | ND |
|  | 12 | PSU5322 | O3:K6 | 2012 | Thailand | 7.76 | ND | ND |
|  | 13 | PSU5325 | O3:K6 | 2012 | Thailand | 2.25 | ND | ND |
|  | 14 | 1884 | O4:K63 | 1996 | USA | 12.20 | ND | ND |
|  | 15 | 1886 | O1:K56 | 1995 | USA | 0.46 | ND | ND |
|  | 16 | 1939 | O4:K12 | 1996 | USA | 0.53 | ND | ND |
|  | 17 | 1926 | O4:K12 | 1996 | USA | 5.44 | ND | ND |
|  | 18 | 1929 | O11:KUT | 1990 | USA | 2.20 | ND | ND |
|  | 19 | 2237 | O4:K11 | 1994 | Bangladesh | 4.32 | ND | ND |
|  | 20 | 2494 | O12:KUT | 1991 | Thailand | 3.63 | ND | ND |
| *tdh^+^ trh*2*^+^* | 1 | 2502 | O1:KUT | 1992 | Thailand | 7.17 | ND | ND |
| (n=18) | 2 | 2542 | O1:K69 | 1886 | Thailand | 12.20 | ND | ND |
|  | 3 | 2508 | O1:K69 | 1993 | Thailand | 6.73 | ND | ND |
|  | 4 | 1990 | O1:KUT | 1994 | India | 16.88 | ND | ND |
|  | 5 | 2443 | O10:K71 | 1987 | Philippines | 14.76 | ND | ND |
|  | 6 | 2554 | O8:K56 | 1994 | Thailand | 3.95 | ND | ND |
|  | 7 | 2759 | O4:K12 | 1995 | Malaysia | 12.81 | ND | ND |
|  | 8 | 2435 | O3:KUT | 1983 | Philippines | 21.05 | ND | ND |
|  | 9 | 2437 | O1:K1 | 1983 | Hong Kong | 1.75 | ND | ND |
|  | 10 | 2439 | O1:K1 | 1984 | Thailand | 11.17 | ND | ND |
|  | 11 | 2440 | O1:K69 | 1985 | Singapore | 5.58 | ND | ND |
|  | 12 | 2458 | O6:K46 | 1983 | Philippines | 4.51 | ND | ND |
|  | 13 | 2463 | O3:KUT | 1986 | Thailand | 18.96 | ND | ND |
|  | 14 | 2472 | O3:K72 | 1989 | Thailand | 2.74 | ND | ND |
|  | 15 | 2475 | O1:KUT | 1989 | Thailand | 15.08 | ND | ND |
|  | 16 | 2496 | O1:KUT | 1992 | Singapore | 4.78 | ND | ND |
|  | 17 | 2498 | O1:KUT | 1992 | Thailand | 3.34 | ND | ND |
|  | 18 | 2503 | O1:KUT | 1992 | Hong Kong | 14.37 | ND | ND |
| *tdh^-^ trh*1*^+^* | 1 | 1927 | O4:K12 | 1990 | USA | 1.49 | 2 | 0.75 |
| (n=15) | 2 | 2182 | O4:K11 | 1981 | Bangladesh | 18.44 | 1 | 18.44 |
|  | 3 | 1895 | O3:K6 | 1985 | Maldives | 19.21 | 1 | 19.21 |
|  | 4 | 2206 | O3:K6 | 1987 | Thailand | 0.89 | 1 | 0.89 |
|  | 5 | 2212 | O3:K6 | 1993 | Thailand | 1.12 | 2 | 0.56 |

| Isolates | No. of isolates | Isolate assigned | Serotype | Year | Country | UP | HA | UP/HA |
| --- | --- | --- | --- | --- | --- | --- | --- | --- |
|  | 6 | 2529 | O5:KUT | 1995 | Thailand | 3.90 | 2 | 1.95 |
|  | 7 | 8705 | O4:K11 | 1985 | Singapore | 1.08 | 1 | 1.08 |
|  | 8 | 2207 | O3:K6 | 1987 | Thailand | 20.79 | 2 | 10.40 |
|  | 9 | PSU2688 | O1:K69 | 2006 | Thailand | 15.67 | 2 | 7.84 |
|  | 10 | PSU1884 | O1:K56 | 2004 | Thailand | 6.82 | 2 | 3.14 |
|  | 11 | PSU77 | O1:K48 | 2003 | Thailand | 17.09 | 2 | 8.55 |
|  | 12 | A927 | O1:K1 | 2010 | Vietnam | 2.21 | 3 | 0.74 |
|  | 13 | PSU393 | O3:KUT | 2005 | Thailand | 9.52 | 2 | 4.76 |
|  | 14 | PSU36 | O3:KUT | 2003 | Thailand | 1.89 | 1 | 1.89 |
|  | 15 | PSU2687 | O4:K53 | 2006 | Thailand | 19.28 | 3 | 6.43 |
| *tdh^-^ trh*2*^+^* | 1 | PSU5256 | O1:KUT | 2012 | Thailand | 8.66 | 1 | 8.66 |
| (n=20) | 2 | PSU5257 | O1:K41 | 2012 | Thailand | 11.17 | 2 | 5.59 |
|  | 3 | PSU5331 | O1:K69 | 2012 | Thailand | 12.04 | 2 | 6.02 |
|  | 4 | 5066 | O1:KUT | 1999 | Thailand | 13.75 | 2 | 6.88 |
|  | 5 | 5279 | O1:K41 | 1999 | Thailand | 10.58 | 1 | 10.58 |
|  | 6 | 1881 | O11:K15 | 1995 | USA | 7.03 | 1 | 7.03 |
|  | 7 | 2226 | O1:K56 | 1983 | Bangladesh | 10.47 | 2 | 5.24 |
|  | 8 | 2293 | O1:K25 | 1986 | Bangladesh | 6.43 | 2 | 3.22 |
|  | 9 | 2357 | O3:KUT | 1977 | Bangladesh | 8.81 | 1 | 8.81 |
|  | 10 | 2374 | O1:K56 | 1980 | Bangladesh | 16.19 | 2 | 8.10 |
|  | 11 | 2380 | O3:KUT | 1980 | Bangladesh | 21.20 | 1 | 21.20 |
|  | 12 | 1919 | O3:K59 | 1991 | USA | 11.83 | 1 | 11.83 |
|  | 13 | 1980 | O1:KUT | 1994 | India | 3.92 | 1 | 3.92 |
|  | 14 | 2149 | O1:KUT | 1982 | Bangladesh | 13.95 | 1 | 13.95 |
|  | 15 | 2174 | O3:K7 | 1982 | Bangladesh | 12.13 | 1 | 12.13 |
|  | 16 | 2185 | O13:KUT | 1981 | Bangladesh | 15.95 | 2 | 7.75 |
|  | 17 | PSU5323 | O1:K25 | 1994 | Thailand | 9.34 | 1 | 9.34 |
|  | 18 | 2351 | O5:KUT | 1977 | Bangladesh | 4.96 | 3 | 1.65 |
|  | 19 | 2388 | O1:KUT | 1980 | Bangladesh | 3.46 | 1 | 3.46 |
|  | 20 | 2368 | O1:KUT | 1980 | Bangladesh | 5.22 | 1 | 5.22 |

**Table S1 Urease production, haemolytic activity and the ratio of urease production to haemolytic activity of all 73 *trh^+^* *V. parahaemolyticus* isolates (continued)**

^a^ Urease production (micromoles of NH_3_ per minute per milligram of protein)

^b^ Haemolytic activity using blood agarose assay

^c^ The ratio of urease production to haemolytic activity (micromoles of NH_3_ per minute per milligram of protein)

^d^ Not determined
