# Supplementary material for: Characterization and CRISPR-based genotyping of clinical trh-positive Vibrio parahaemolyticus
Source: Gut Pathog. 2018 Nov 13;10:48. doi: 10.1186/s13099-018-0275-4 (PMC6233571; doi:10.1186/s13099-018-0275-4)
Supplement: Supplementary file 2 — Additional file 2: Table S2. Foreign genetic element similar to spacers using CRISPR targets analysis. [file 13099_2018_275_MOESM2_ESM.docx]

Additional file 2

**Table S2 Foreign genetic element similar to spacers using CRISPR targets analysis**

| Spacer groups | Spacers | [Nucleotide sequence of](https://link.springer.com/content/pdf/10.1007%2FBF00019953.pdf)spacers (bp) | Similar genetic elements/  hypothetical proteins | Accession number |
| --- | --- | --- | --- | --- |
| SP1 | PSU5323-s1 | TCATTCTCACGATCTAATTACAGTTGGCAC (30) | *Vibrio alginolyticus* strain K06K5 plasmid | NZ_CP017898.1 |
|  | PSU5256-s1 | TCATTCTCACGGATCTAATAACAGTTGGTCAC (32) | *-Escherichia coli* strain Ecol_AZ159 and EQ011 plasmid**s**  *-Enterobacter cloacae* strain 704SK10 and R11 plasmid**s**  *-Klebsiella pneumoniae* strain CAV1193-258 and CAV1344-250 plasmids | NZ_CP019007.1 and NC_023315.1  NZ_CP022149.1 and NZ_CP019840.1  NZ_CP013323.1 and NZ_CP011622.1 and NZ_CP011623.1 |
|  | PSU4921-s1, PSU5107-s1, PSU5296-s1, 2443-s1, 2463-s1 | TCATTCTCACGATCTAATTACAGTTGGTCAC (31) | *Vibrio alginolyticus* strain K06K5 and K04M3 plasmids | NZ_CP017898.1 and NZ_CP017909.1 |
|  | PSU5322-s1, PSU5331-s1 | TCATTCTCACGATCTAATTACAGTTGGCAC (30) | - | - |
|  | 2435-s1 | TCATTCTCACGGATCTAATTACAGTTGGTCAC (32) | - | - |
| SP2 | 2475-s1 | CTCCAACTAAGTTTTCTGTAGCTTCATCATT (32) | *Vibrio alginolyticus* strain K04M3, K08M3 and K04M5 plasmids | NZ_CP017898.1, NZ_CP 017915.1 and NZ_CP017901.1 |
| SP3 | PSU5264-s1 | TGCAGACAAACAAAGAGGCATCGACGAGTGCAT (33) | *Vibrio alginolyticus* strain K04M3 and K04M5 plasmids | NZ_CP017898.1 and NZ_CP017901.1 |
|  | 1884-s1, 1990-s1 | TGCAGACAAACAAAGAGCCATCGACGAGTGC (31) | *Vibrio alginolyticus* strain K04M3, K08M3 and K04M5 plasmids | NZ_CP017898.1, NZ_CP 017915.1 and NZ_CP017901.1 |
| SP4 | PSU5105-s1, PSU5106-s1 | GAGATACCACAGGCTCAAGCAGATGCTAACAG (31) | *Vibrio alginolyticus* strain K06K5 and K04M3 plasmids | NZ_CP017898.1 and NZ_CP017909.1 |
| SP5 | PSU5256-s2 | AGTCGGTCAACTGAGAATACGTTGTTGCCAA (31) | - | - |
| SP6 | PSU4921-s2, PSU5105-s2, PSU5106-s2, PSU5107-s2, PSU5296-s2, PSU5322-s2, 2443-s2, 2463-s2 | TCATTCTCACGATCTAATTACAGTTGGTCAC (31) | *Vibrio alginolyticus* strain K06K5 and K04M3 plasmids | NZ_CP017898.1 and NZ_CP017909.1 |
|  | PSU5323-s2, PSU5331-s2, 2435-s2 | AGTCGGTCAACTGAGAATCGTTTGTTGCCAA (31) | - | - |
